# Continuing Professional Development – Radiation Therapy

**DOI:** 10.1002/jmrs.70051

**Published:** 2025-12-21

Maximise your continuing professional development (CPD) by reading the selected article and answering the five questions. Please remember to self‐claim your CPD and retain your supporting evidence. Answers will be available via the QR code and published in JMRS—Volume 73, Issue 4, December 2026.

## Development and Evaluation of an Electronic Patient‐Reported Outcome Platform for Children Undergoing Radiation Therapy

Mikaela Doig, Andrew Cunningham, Victoria Bedford, Hien Le, Matthew O'Connor, Sophie Jessop, Eva Bezak, Nayana Parange, Amanda Hutchinson, Peter Gorayski, Michala Short, https://doi.org/10.1002/jmrs.70025.

1. Which of the following would constitute a patient‐reported outcome?
  - A practitioner documents a child's pain score based on observation.
  - A parent reports their child's fatigue on the child's behalf.
  - A child completes an online questionnaire rating their own fatigue.
  - A clinician records the child's white blood cell count after treatment.
2. How can an electronic patient‐reported outcome measure (ePROM) platform help reduce common barriers to implementing PROMs in clinical practice?
  - It removes the need to validate questionnaires.
  - It requires users to have advanced technical skills.
  - It enables automated scoring, real‐time data availability and smoother workflow integration.
  - It eliminates all patient burden.
3. What methodology was used to develop and assess the ePROM platform in the study?
  - User‐centred design with feedback and iterative testing
  - A randomised controlled trial
  - Modifying an existing platform without stakeholder input
  - A cross‐sectional survey
4. What was reported during the beta‐testing phase of the platform?
  - Most participants found it unusable
  - It was too complex for children, so paper forms were used
  - No refinements were suggested
  - It was intuitive and child‐friendly, requiring only minor adjustments
5. According to the study, what is a key consideration for future clinical use of the ePROM platform?
  - Handling parental access to a child's quality of life data with care
  - Prioritising parent proxy input over the child's self‐report
  - Requiring all patients to use the platform, regardless of context
  - Limiting healthcare professionals' access to reports due to time constraints

## Answers

Scan this QR code to find the answers.
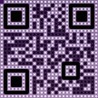

